# The Predominant Protective Effect of Tianeptine Over Other Antidepressants in Models of Neuronal Apoptosis: The Effect Blocked by Inhibitors of MAPK/ERK1/2 and PI3-K/Akt Pathways

**DOI:** 10.1007/s12640-013-9430-3

**Published:** 2013-10-09

**Authors:** D. Jantas, S. Krawczyk, W. Lason

**Affiliations:** Department of Experimental Neuroendocrinology, Institute of Pharmacology, Polish Academy of Sciences, Smetna 12, 31-343 Kraków, PL Poland

**Keywords:** Apoptosis, Neuroprotective drugs, Antidepressant drugs, Primary neuronal and glia cell cultures, SH-SY5Y cells, PI3-K/Akt, MAPK/ERK1/2, Necrostatin-1

## Abstract

Tianeptine (Tian) possesses neuroprotective potential, however, little is known about the effect of this drug in models of neuronal apoptosis. In the present study, we aimed (1) to compare the neuroprotective capacities of some antidepressants (ADs) in the models of staurosporine (St)- and doxorubicin (Dox)-evoked cell death, activating the intracellular and the extracellular apoptotic pathway, respectively; (2) to identify the Tian-modulated steps underlying its neuroprotective action; (3) to test the effect of various ADs against Dox-evoked cell damage in glia cells. Primary neuronal and glia cell cultures and retinoic acid-differentiated human neuroblastoma SH-SY5Y (RA-SH-SY5Y) cells were co-treated with imipramine, fluoxetine, citalopram, reboxetine, mirtazapine or Tian and St or Dox. The data showed the predominant neuroprotective effect of Tian over other tested ADs against St- and Dox-induced cell damage in primary neurons and in RA-SH-SY5Y cells. This effect was shown to be caspase-3-independent but connected with attenuation of DNA fragmentation. Moreover, neuroprotection elicited by Tian was blocked by pharmacological inhibitors of MAPK/ERK1/2 and PI3-K/Akt signaling pathways as well by inhibitor of necroptosis, necrostatin-1. Interestingly, the protective effects of all tested ADs were demonstrated in primary glia cells against the Dox-evoked cell damage. The obtained data suggests the glial cells as a common target for protective action of various ADs whereas in relation to neuronal cells only Tian possesses such properties, at least against St- and Dox-induced cell damage. Moreover, this neuroprotective effect of Tian is caspase-3-independent and engages the regulation of survival pathways (MAPK/ERK1/2 and PI3-K/Akt).

**Electronic supplementary material:**

The online version of this article (doi:10.1007/s12640-013-9430-3) contains supplementary material, which is available to authorized users.

## Introduction

Tianeptine (7-[(3-chloro-6-methyl-5,5-dioxo-11*H*-benzo[c][2,1]benzothiazepin-11-yl)amino]heptanoic acid) belongs to the group of atypical antidepressants (ADs) with a unique mechanism of action which is still not fully understood (McEwen and Olie 2005; McEwen et al. 2010). However, in contrast to other ADs, tianeptine (Tian) has been reported to increase neuronal serotonin (5-HT) reuptake, but this phenomenon is rather not responsible for the clinical efficacy of this drug (Fattaccini et al. 1990; McEwen et al. 2010; Mennini et al. 1987). In fact, more specific studies clearly showed that acute and long-term administration of Tian did not elicit any marked alterations in extracellular 5-HT levels in corticolimbic structures of conscious rats (Malagie et al. 2000; Pineyro et al. 1995). It is well documented that Tian mediates the AD effect via the regulation of HPA axis and normalization of the glutamatergic system (McEwen et al. 2010). The latter is connected with the ability of Tian to modulate (1) glutamate transmission via induction of serine phosphorylation at AMPA receptors which positively regulates glutamate transmission and plasticity at excitatory synapses (Kole et al. 2002; Qi et al. 2009; Svenningsson et al. 2007; Szegedi et al. 2011; Zhang et al. 2012b) and (2) glutamate turnover as demonstrated by the inhibiting effect of Tian on chronic restraint stress-induced changes in glutamate transporter EAAT2 (GLT1) expression on glial cells (Reagan et al. 2004) and stimulating properties on glutamate efflux and vGLUT2 expression in central amygdalar complex of rats subjected to repeated stress (Piroli et al. 2013).

Many experimental studies and clinical observations underline the engagement of degenerative processes of neuronal and glia cells in the pathomechanism of depression (Catena-Dell’Osso et al. 2013; Cobb et al. 2013; Fuchs et al. 2004; McKernan et al. 2009; Rajkowska 2002; Rajkowska and Miguel-Hidalgo 2007). On the other hand, clinically used AD drugs exhibited neuroprotective properties in various in vitro and in vivo models of neuronal injury although usually chronic treatment was required to achieve beneficial effects (Drzyzga et al. 2009; McEwen et al. 2010; McKernan et al. 2009). Regarding the neuroprotective action of Tian, this drug has been shown to possess anti-apoptotic action after chronic treatment (50 mg/kg p.o., 28 days) in the psychosocial stress model in tree shrews (Lucassen et al. 2004) as well prevented the morphological changes in the brain of chronically stressed rats (Della et al. 2012a). Other reports provided data on the neuroprotective action of Tian in in vitro (NMDA, hypoxia) and in vivo (ibotenic acid) models of excitotoxicity, although only when the cell-damaging agents were combined with interleukin-1β treatment. The beneficial effect of Tian in the above models was explained by anti-inflammatory properties of this drug (Plaisant et al. 2003), which was also confirmed by a normalizing effect of Tian on production of pro- and anti-inflammatory cytokines after lipopolysaccharide (LPS) treatment in rat hypothalamus (Castanon et al. 2004). Recently, it has been shown that Tian affords protection not only to neurons but also to glia cells. Studies performed on astroglial cell lines demonstrated the attenuating effect of Tian (≥10 μM) on apoptosis induced by glycoprotein 120 (gp120) (Janda et al. 2011).

It should be underlined that in most studies on neuroprotective potential of various class of ADs, only one or a few of them were tested in parallel in one study. However, it seems that for reliable comparison of neuroprotective effectiveness of various ADs, the drugs should be tested parallelly under the same experimental settings. Secondly, there is still an open question of how far the protection mediated by various ADs (including also Tian) is neuron-specific or whether it engages also other cells (e.g., glia cells). Regarding the neuroprotective capacities of Tian, there is still a lack of data on the effects of this drug in models of neuronal injury not directly connected with glutamate overactivation (excitotoxicity), especially in models of apoptosis. In the present study, we aimed to test the effect of various ADs belonging to different pharmacological classes [tricyclic AD drug-imipramine (Imi), selective serotonin reuptake inhibitors (SSRIs): fluoxetine (Flu) and citalopram (Cit), selective noradrenaline reuptake inhibitor—reboxetine (Reb) and atypical ADs—mirtazapine (Mirt) and Tian]. It should be mentioned here that apart from the modulation of the monoaminergic system, chronic ADs treatment has been shown to selectively increase expression of neurotrophins (BDNF) and plasticity-related proteins (PSA-NCAM, pCREB, GAD-43) in the hippocampus and prefrontal cortex of the rat (McKernan et al. 2009).

In the present study, we tested the chosen ADs in a wide concentration range (0.001–10 μM) in two typical models of cellular apoptosis. We used two chemical agents for induction of mitochondrial (staurosporine, St) and extracellular (doxorubicin, Dox) apoptotic pathway in mouse primary cortical neurons and in retinoic acid-differentiated human neuroblastoma SH-SY5Y (RA-SH-SY5Y) cells. We also tested the effect of ADs on cell death induced by Dox in mouse primary glia cells. The results may provide further information on which ADs exert the best neuroprotective effect in models of neuronal apoptosis directly unrelated to the glutamatergic overactivation (Jantas et al. 2009; Jantas and Lason 2009).

## Methods

### Reagents and Antibodies

Antidepressant drugs: imipramine hydrochloride (Sigma-Aldrich Chemie GmbH, Germany), fluoxetine hydrochloride (Farmacom, Poland), citalopram (Lundbeck, Denmark), reboxetine (Pharmacia, Italy), mirtazapine (Organon, The Netherlands), tianeptine sodium (Servier, France).

Neurobasal A medium, Dulbecco’s modified Eagle’s medium (DMEM), fetal bovine serum (FBS), supplement B27 were from Gibco (Invitrogen, Paisley, UK). The Cytotoxicity Detection Kit, BM Chemiluminescence Western Blotting Kit and In Situ Cell Death Detection Kit Fluorescein were from Roche Diagnostic (Mannheim, Germany). Primary antibodies: anti-MAP-2 (sc-51669), antiphospho-Tyr^204^ ERK 1/2 (pERK, sc-7383), anti-ERK2 (sc-474), anti-spectrin α IIsc-48382), protein markers and appropriate secondary antibodies were from Santa Cruz Biotechnology Inc. (CA, USA). All other reagents were from Sigma (Sigma-Aldrich Chemie GmbH, Germany).

### Neuronal Cell Cultures

The experiments were conducted on primary cultures of mouse cortical neurons and on neuronal differentiated human neuroblastoma SH-SY5Y cells as described previously (Jantas et al. 2008; Jantas-Skotniczna et al. 2006).

The protocol for generation of the primary neuronal cultures was concordant with local and international guidelines on the ethical use of animals. Neuronal tissues were taken from Swiss mouse embryos at 15/16th day of gestation and were cultured essentially as described previously (Jantas-Skotniczna et al. 2006). The isolated cortical cells were suspended in Neurobasal medium containing penicillin (0.06 μg/ml) and streptomycin (0.1 μg/ml), supplement B27 without antioxidants and were plated at a density of 1.5 × 10^5^ cells/cm^2^ onto poly-ornithine (0.05 mg/ml)-coated multi-well plates. This procedure typically yields cultures that contain >90 % neurons and <10 % supporting cells as verified by immuncytochemistry (not shown). The cultures were then maintained at 37 °C in a saturated humidity atmosphere containing 95 % air and 5 % CO_2_ for 7 days prior to experimentation and culture medium was exchanged every 2 days.

The human SH-SY5Y neuroblastoma cells (ATCC), passages 5–20 were grown in DMEM supplemented with 10 % heat-inactivated FBS and 100 units/ml of penicillin and 100 μg/ml of streptomycin as described previously (Jantas et al. 2008). Cells were maintained at 37 °C in a saturated humidity atmosphere containing 95 % air and 5 % CO_2_. After reaching an 80 % confluence, cells were seeded onto appropriate multi-well plates at a density of 4 × 10^4^ cells/cm^2^ and were differentiated to a neuronal phenotype for 7 days by adding retinoic acid (RA) to the culture medium at a final concentration of 10 μM. The culture medium with RA was changed three times during the period of differentiation. One day before the experimental treatment of RA-differentiated SH-SY5Y cells, the culture medium was replaced by DMEM containing antibiotics and 1 % FBS.

### Glia Cell Cultures

Cortical glia cells were prepared from 2-day-old Albino Swiss mice according to the protocol described by Johann et al. (2008) with some modifications. Briefly, cerebral cortices were dissected, meninges were removed and tissues were minced separately into small pieces, then digested with trypsin (0.1 % for 30 min at 37 °C), triturated in the presence of 20 % fetal bovine serum and DNAse I (150 Kunitz units/ml), and finally centrifuged for 5 min at 100×*g*. Cells were resuspended in DMEM containing 20 % FBS and 100 units/ml of penicillin and 100 μg/ml of streptomycin. Finally, cells were seeded into 75-cm^2^ culture bottles coated with poly-ornithine (0.05 mg/ml) and grown at 37 °C in a humidified incubator with 5 % CO_2_. Culture medium initially contained 20 % of FBS and after 4 days was replaced with medium containing 10 % FBS and was exchanged twice a week. Cells after reaching about 80–90 % confluence were trypsinized and replated at a lower density. This procedure was repeated three times and after the last trypsinization cells were seeded on poly-ornithine-coated 96-well plates. Four- week-old glia cell cultures were used for drug testing. Three days before drug exposure culture medium was replaced with the culture medium used for cortical neurons (Neurobasal medium, antibiotics, and B27 supplement) in order to reduce cell proliferation and to achieve similar experimental conditions for drug treatment as in primary neurons. One day before the cell treatment, Neurobasal culture medium was replaced with fresh one. The obtained glia cultures are characterized by about 90 % homogeneity of glial fibrillary acidic protein (GFAP)-positive cells with no neuronal (MAP-2-positive cells) contamination (not shown).

### Immunocytochemistry

The purity of primary neuronal and glia cultures and the morphological changes in cortical neurons after a particular drug treatment were determined by immunocytochemistry as described by Singh et al. (2010). At particular DIV (7th for neuronal and 4 weeks for glia cells) cells were washed with pre-warmed phosphate-buffered saline (PBS), fixed for 20 min at RT with 4 % paraformaldehyde and washed twice with PBS. Next the cells were permeabilized with PBS-containing 0.25 % Triton X-100 (PBS-TX-100) for 15 min after which the blocking was performed in the presence of 5 % normal goat serum in PBS-TX-100 at room temperature for 60 min. Primary antibodies against neuronal (anti-MAP-2, 1:250) and glia (anti-GFAP, 1:500) markers were added and incubated with cells for the next 60 min at RT. Subsequently, cells were washed three times with PBS and incubated for 60 min at RT in the presence of secondary antibodies: Alexa Fluor^®^488 labeled goat anti-mouse and Alexa Fluor^®^568 labeled goat anti-rabbit IgG (Invitrogen, USA) diluted 1:500 in PBS. Cells were washed with PBS three times for 5 min and mounted with ProLong^®^Gold antifade reagent (Invitrogen, USA). Cells were examined using a fluorescence AxioObserver microscope (Carl Zeiss, Germany) equipped with the software Axiovision 3.1 at excitation wavelengths of 470 nm (Alexa Fluor^®^488) and 555 (Alexa Fluor^®^568).

### Cell Treatment

Seven days in vitro (DIV) cortical neurons were co-treated with Tian (0.001–10 μM) and St (0.5 μM) or Dox (0.5 μM) for 14–48 h. RA-SH-SY5Y cells were incubated with Tian (0.01–10 μM) and St (0.5 μM) or Dox (1 μM) for 14–48 h. The effective concentrations and time of exposure to St and Dox were chosen on the basis of our previous reports where these pro-apoptotic factors evoked about 50 % reduction in cell viability of cortical neurons and RA-SH-SY5Y cells (Jantas et al. 2008, 2009; Jantas and Lason 2009; Jantas-Skotniczna et al. 2006). For comparative studies between the effects of Tian with other ADs, cortical neurons and RA-SH-SY5Y cells were co-treated with Imi, Flu, Cit, Reb, or Mirt at concentrations from 0.01 to 10 μM and St or Dox. In glia cell cultures, we also tested the protective effect of Tian and other ADs (Imi, Flu, Cit, Reb, Mirt) at concentrations 0.01–10 μM against cell damage induced by Dox (1 μM for 48 h). An involvement of PI-3 K/Akt and MAPK/ERK/1/2 pathways as well as necroptosis in Tian-mediated neuroprotection was tested by using specific inhibitors of these pathways: LY294002 (10 μM), U0126 (10 μM) and necrostatin-1 (10 μM) which were added 30 min before treatment of neuronal cells with Tian and St or Dox. The caspase-3 inhibitor (Ac-DEVD-CHO, 10 μM) was added to cells 20 min before treatment of cells with St or Dox. St (1 mM), Ac-DEVD-CHO (10 mM), LY 294002 (10 mM), necrostatin-1 (100 mM), and U0126 (10 mM) stock solutions were prepared in dimethyl sulfoxide. Mirt stock solution (10 mM) was prepared in distilled water acidified with few drops of 0.1 M HCl followed by pH adjustment to 7.15 with 0.1 M NaOH. Dox, Imi, Flu, Cit, Reb, and Tian were dissolved in distilled water. The chemicals were present in cultures at a final concentration of 0.1 % and vehicle-treated cells were treated with a relevant solvent.

### Measurement of Lactate Dehydrogenase (LDH) Release

In order to estimate cell death, the level of lactate dehydrogenase (LDH) released from damaged cells into culture media was measured after 24 and 48 h of treatment of cells with ADs and St or Dox, respectively, as described previously (Jantas-Skotniczna et al. 2006). Cell-free culture supernatants were collected from each well and incubated with the appropriate reagent mixture according to the supplier’s instructions (Cytotoxicity Detection Kit, Roche) at RT for 20 min. The intensity of red color formed in the assay and measured at a wavelength of 490 nm was proportional to LDH activity and to the number of damaged cells. Absorbance of blanks, determined as no-enzyme control, has been subtracted from each value. The data were normalized to the activity of LDH released from vehicle-treated cells (cortical neurons and glia cells) or toxin-treated cells (RA-SH-SY5Y cells) and expressed as a percent of the control ± SEM or a percent of toxin-induced ± SEM established from *n* = 5 wells per one experiment from three separate experiments.

### MTT Reduction Assay

Cell viability assessment was done after 24–48 h of treatment of cortical neurons, glia cells, and RA-SH-SY5Y cells with particular chemicals. Cell damage was quantified using a tetrazolium salt colorimetric assay with 3-[4,5-dimethylthiazol-2-yl]-2,5-diphenyltetrazolium bromide (MTT) as described previously (Jantas et al. 2011). The absorbance of each sample was measured at 570 nm in a 96-well plate-reader (Multiscan, Labsystem). The data after subtraction of blanks (absorbance of cells without MTT) were normalized to the absorbance in the vehicle-treated cells (100 %) and expressed as a percent of the control ± SEM established from *n* = 5 wells per one experiment from three separate experiments.

### CalceinAM Staining of Viable Cells

In order to morphologically assess the changes in cell viability in cortical neurons after treatment with Tian and pro-apoptotic agents, we employed a cell-permeable CalceinAM dye according the protocol described previously (Jantas and Lason 2009). The cells were evaluated using an inverted fluorescence microscope (AxioObserver, Carl Zeiss) with excitation wavelength 480 nm (green fluorescence).

### Identification of Pyknotic Nuclei by Hoechst 33342 Staining

In order to visually assess the changes in DNA structure after cell treatment with pro-apoptotic factors and Tian in cortical neurons and in RA-SH-SY5Y cells, Hoechst 33342 staining was applied as described previously (Jantas et al. 2011). That dye binds to double-stranded DNA and can be used to visualize highly compacted chromatin of fragmented pyknotic cell nuclei. Images were recorded using an inverted fluorescence microscope (AxioObserver, Carl Zeiss, Germany) with excitation wavelength 350 nm (blue fluorescence). Uniformly stained nuclei were scored as healthy, viable nuclei while those with condensed or fragmented nuclei were identified as damaged one. The number of cells with nuclei having normal and changed (condensed or fragmented) morphology was counted in six randomly chosen fields per a cover slip (150–200 cells); two cover slips per condition from three separate experiments were evaluated. The data were calculated as a percentage of damaged nuclei compared to the total number of cells per one field and presented in histograms as the mean ± SEM.

### Identification of Apoptotic Nuclei by TUNEL Method

In order to determine DNA fragmentation in apoptotic cells, the terminal deoxynucleotidyl transferase (TdT)-mediated dUTP nick-end labeling (TUNEL) technique was applied using the In Situ Cell Death Detection Kit Fluorescein (Roche Diagnostic) as described previously (Jantas et al. 2011). The cortical neurons after Tian and St (24 h) or Dox (36 h) exposure were washed with PBS, fixed with 4 % paraformaldehyde for 20 min and washed two times with PBS. Subsequently, the cells were treated with 0.1 % sodium citrate/0.1 % Triton X-100 for 2 min on ice, and incubated with TUNEL reaction mixture for 60 min at 37 °C. After washing, the TUNEL-labeled nuclei (green points) were examined under excitation 470 nm using inverted fluorescence microscope (AxioObserver, Carl Zeiss). Apoptotic nuclei were counted on recorded images from six randomly chosen fields per a cover slip, two cover slips per condition from three separate experiments and are shown as the mean ± SEM of apoptotic nuclei per one field.

### Assessment of Caspase-3 Activity

The caspase-3 protease activity assay in the cortical neuronal samples treated with St (0.5 μM) and Dox (0.5 μM) for 14 and 24 h, respectively, was performed on 96-well plates using colorimetric substrate preferentially cleaved by caspase-3, AcDEVD-pNA (*N*-acetyl-asp-glu-val-asp *p*-nitro-anilide) as described previously (Jantas-Skotniczna et al. 2006). As a marker of assay specificity, the cell-permeable caspase-3 inhibitor, AcDEVD-CHO (10 μM) was added to St- or Dox-treated cells during cell treatment. The amounts of *p*-nitroanilide cleaved by caspase-3 were monitored continuously over 60 min with a plate-reader (Multiscan, Labsystems) at 405 nm. Absorbance of blanks, determined as no-enzyme control, has been subtracted from each value. The data were normalized to the absorbance of vehicle-treated cells (100 %) and expressed as percent of absorbance ± SEM established from *n* = 5 wells per experiment from three separate experiments.

The measurements of caspase-3 activity in RA-SH-SY5Y cells were performed in the same lysis buffer as described for primary neurons (Jantas-Skotniczna et al. 2006) but with some modifications. Cells were cultured in six-well plate and after drug exposure (Tian and St or Dox for 12 and 24 h, respectively), the culture medium was removed, and cells were washed with cold PBS and stored at −20 °C until measurement (no more than 1 week). Next, the cells were thawed on ice and lysed in 150 μl of ice-cold Caspase Assay Buffer (50 mM HEPES, pH 7.4, 100 mM NaCl, 0.1 % CHAPS, 1 mM EDTA, 10 % glycerol, and 10 mM dithiothreitol) lysis buffer supplemented with 10 μg/ml of each leupeptin and pepstatin A for 15 min at 4 °C. Next, the lysed cells were scraped and collected in separate tubes and centrifuged at 16,000×*g* for 20 min at 4 °C. The supernatants were used for determination of caspase-3 activity by the usage of fluorometric substrate Ac-DEVD-AMC (Promega), according to which the amount of fluorochrome 7-amino-4-methyl coumarin (AMC) is released from the substrate (Ac-DEVD-AMC) upon cleavage by caspase-3-like enzymes. A yellow-green fluorescence produced by free AMC is proportional to the caspase-3 activity present in the sample. Cell lysates (50 μl) were incubated with Ac-DEVD-AMC (50 μM) for 60 min at 37 °C in the absence and presence of a specific caspase-3 inhibitor (Ac-DEVD-CHO; 10 μM) and the fluorescence was measured with a plate-reader (Infinite^®^ M1000 PRO, Tecan, Switzerland) at 360 nm excitation and 460 nm emission wavelengths. The measurement was performed in triplicates and mean RFU (relative fluorescence units) were calculated per mg of protein for each experimental sample. The protein concentration in cell lysates was determined with the bicinchoninic acid protein assay kit (BCA1, Sigma). Data were presented as the mean RFU/mg protein ± SEM established from *n* = 3 wells per one experiment from three independent experiments.

### Western Blotting Analysis

For Western blot analysis, cortical neurons were cultured in six-well plates coated with poly-ornithine (0.05 mg/ml) and after 7 DIV were treated for 6 and 18 h with Tian (0.1 and 10 μM) and St or Dox. For preparation of the whole cell lysates, cells were washed with ice-cold PBS and harvested and lysed with ice-cold RIPA buffer (150 mM NaCl, 1.0 % IGEPAL CA-630, 0.5 % sodium deoxycholate, 0.1 % SDS, 50 mM Tris, pH 8.0) in the presence of a cocktail containing protease inhibitors. For measurement of phosphorylated forms of kinases (pERK), the lysis buffer was enriched with heat-activated sodium orthovanadate and phosphatase inhibitors (cocktail I and II). Cell lysates were centrifuged at 20,000×*g* for 15 min at 4 °C and the supernatants were stored at −20 °C until further use. Protein amounts were determined with the BCA method and equal amount of proteins was denatured in a modified Laemmli sample buffer (0.25 M Tris–HCl pH 6.8, 10 % SDS, 40 % glycerol, 10 % 2-mercaptoethanol, 0.5 % bromophenol blue) and boiled for 3 min. An equal amount of protein from experimental groups was separated on 10 % SDS–polyacrylamide gel and transferred onto a PVDF membrane. Membranes were blocked for 1 h with 5 % nonfat milk in TBS-T (Tris-buffered solution pH 7.5/0.005 % Tween 20) and incubated overnight with primary antibodies diluted at 1:500 (spectrin α II), 1:1000 (pERK) and 1:2,000 (ERK2) in 1 % nonfat milk in TBS-T. The amount of ERK2 was determined on the same membrane on which the level of pERK and spectrin α II of were measured by stripping and reprobing the membrane as described previously (Jantas et al. 2011). The primary antibody reaction was followed by 1 h incubation with relevant secondary antibodies connected with horseradish peroxidase. Immunocomplexes were detected using an enhanced chemiluminescence detection system (Roche) and band intensities were determined by densitometric analysis of immunoblots (Fuji Film Las 4000). MultiGauge v.3 Software was used for quantification of Western blot signals. Data from duplicate determinations in three independent experiments were normalized to ERK2 level in particular samples and were shown as fold of control (mean ± SEM).

### Data Analysis

Data after normalization were analyzed using the Statistica software (StatSoft Inc., Tulsa, OK, USA). The analysis of variance (one-way ANOVA) and post-hoc Tukey’s test for multiple comparisons were used to show statistical significance with assumed *p* < 0.05.

## Results

### Tianeptine Protects Neuronal Cells Against Cell Death Induced by Pro-apoptotic Factors

**Fig. 1:**
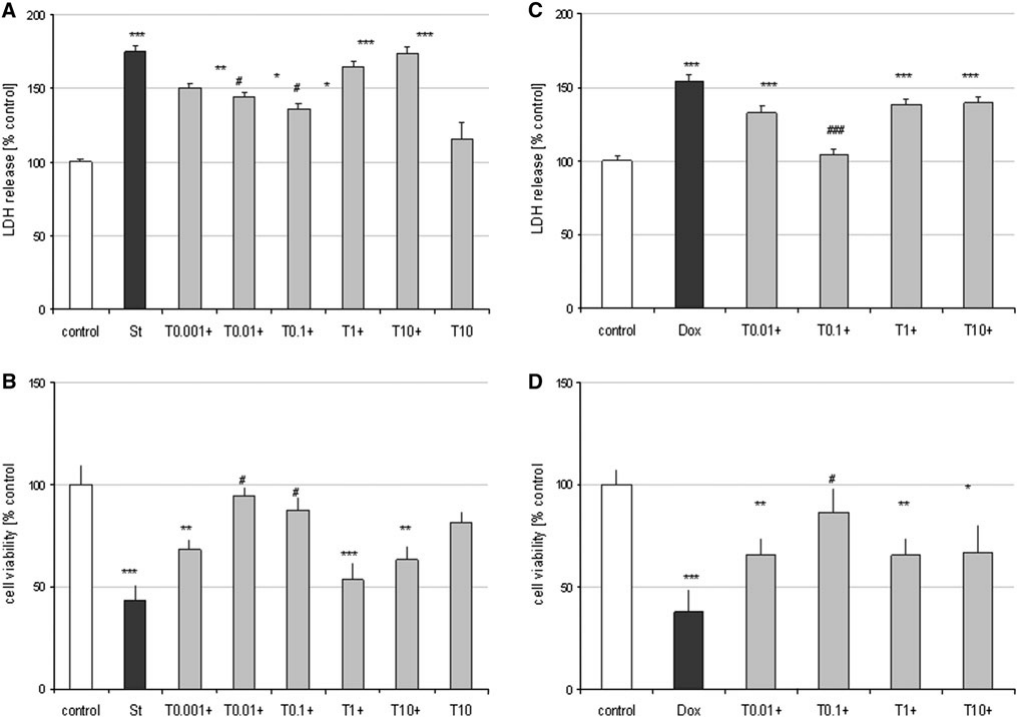
Tianeptine is neuroprotective in two models of neuronal apoptosis in cultured primary neurons. **a** and **b** 7 DIV cortical neurons were co-treated with tianeptine (T: 0.001–10 μM) and staurosporine (St; 0.5 μM) for 24 h. Next, LDH level (**a**) was measured in the culture medium from the treated cells and cell viability was estimated in cells via MTT reduction method (**b**). **c** and **d** 7 DIV cortical neurons were co-treated with tianeptine (T:0.01–10 μM) and doxorubicin (Dox, 0.5 μM) for 48 h. Next, LDH level (**c**) was measured in the culture medium from the treated cells and cell viability was estimated in cells via MTT reduction method (**d**). The data from the biochemical tests were normalized to the absorbance in the vehicle-treated cells (100 %) and expressed as a percent of the control ± SEM established from *n* = 5 wells per one experiment from three separate experiments. *p < 0.05, **p < 0.01, and ***p < 0.001 versus vehicle-treated cells; ^#^ *p* < 0.05 and ^###^ *p* < 0.001 versus St/Dox-treated cells

**Fig. 2:**
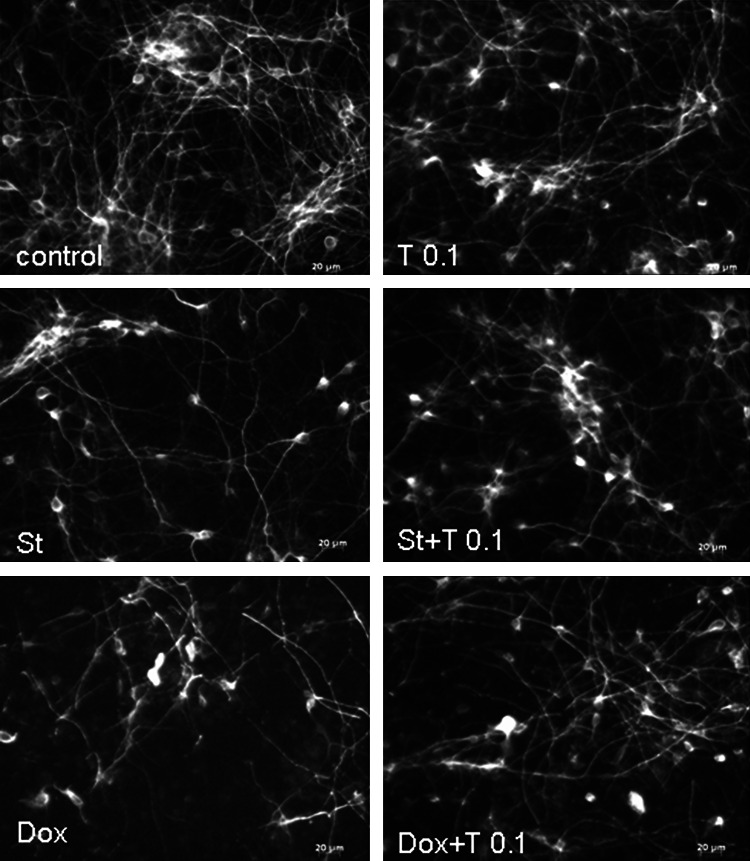
Tianeptine prevents the reduction in the number of MAP-2 positive cells in staurosporine (St)- and doxorubicin (Dox)-treated cortical neurons. Microphotographs from MAP-2 immunofluorescence of cortical neurons treated with tianeptine (T: 0.1 μM) and St (0.5 μM) or Dox (0.5 μM) for 24 and 36 h, respectively. *Photos* were recorded using a inverted fluorescence AxioObserver microscope (Carl Zeiss, Germany) equipped with the software Axiovision 3.1 at excitation wavelengths of 470 nm (Alexa Fluor^®^488)

Cortical neurons at 7 DIV are a suitable model for studies of apoptotic processes evoked by pro-apoptotic factors activating mitochondrial (St) and extracellular (Dox) pathways of apoptosis as we reported previously (Jantas et al. 2009; Jantas-Skotniczna et al. 2006). Tian when given alone (0.01–10 μM) had no effect on cell viability of cortical neurons measured by LDH release and MTT reduction assays (not shown). However, Tian at low concentrations (0.01 and 0.1 μM) partially attenuated the St-induced LDH release (by about 30 % of control value) and increased cell viability by about 45–50 % (Fig. 1a, b). Tian (0.1 μM) was also protective against Dox-evoked cell death by reducing the changes in LDH release and MTT reduction tests almost to the level found in vehicle-treated cells (Fig. 1c, d). The neuroprotective effects of Tian found in biochemical tests was confirmed by live staining of cortical neurons with CalceinAM dye (Sup. Fig. 1) as well as by anti-MAP-2 immunocytochemistry of fixed neurons (Fig. 2).

The neuroprotective effects of Tian was also demonstrated in RA-SH-SY5Y cells where this drug attenuated the St (Tian 0.01 and 0.1 μM)- and Dox (Tian 0.1 μM)-induced LDH release and increased cell viability by about 30–40 % (Sup. Fig. 2).

### Lack of Neuroprotective Effects of Other ADs in the St and Dox Models of Neuronal Apoptosis

In the next part of our study, we compared the neuroprotective effect of Tian with other ADs. During 24 h incubation with Imi, Cit, Reb, Mirt at concentrations from 0.01 to 10 μM, we did not observe any detrimental effects of the tested drugs given alone on cell viability (Table 1). Only Flu at a concentration of 10 μM when given alone significantly reduced (by about 15 %) cell viability and also increased the cell death (by about 20 %) induced by St and Dox (Table 1). However, we did not observe any neuroprotective effects of Imi, Flu, Cit, Reb, Mirt (0.01–10 μM) against St- or Dox-evoked cell damage (Table 1).

**Table 1:** The effects of antidepressants on St- and Dox-evoked cell death in 7 DIV cortical neurons

	Cell viability—St (% control)	Cell viability—Dox (% control)
Control	100.00 ± 1.20	99.93 ± 3.08
Imi 10	89.53 ± 3.75	89.48 ± 3.24
Flu 10	85.96 ± 5.10*	68.24 ± 4.63***
Cit 10	92.15 ± 3.50	104.01 ± 4.17
Reb 10	100.38 ± 7.26	93.78 ± 4.07
Mirt 10	95.43 ± 3.77	117.09 ± 8.21
St/Dox	66.93 ± 1.20***	63.66 ± 2.93***
+Imi 0.01	71.58 ± 5.07	67.02 ± 6.67
+Imi 0.1	78.68 ± 9.16	66.41 ± 5.31
+Imi 1	66.33 ± 3.71	72.16 ± 9.75
+Imi 10	62.61 ± 3.37	61.68 ± 3.81
+Flu 0.01	64.83 ± 6.03	78.74 ± 8.67
+Flu 0.1	64.58 ± 5.33	73.68 ± 6.55
+Flu 1	62.65 ± 4.42	65.13 ± 5.47
+Flu 10	46.57 ± 3.40^#^	35.28 ± 4.86^###^
+Cit 0.01	80.83 ± 8.15	69.47 ± 6.68
+Cit 0.1	78.89 ± 3.91	64.90 ± 7.15
+Cit 1	69.74 ± 3.46	70.10 ± 9.57
+Cit 10	65.09 ± 3.79	66.22 ± 2.92
+Reb 0.01	76.92 ± 6.58	70.77 ± 6.66
+Reb 0.1	72.67 ± 3.27	74.83 ± 7.07
+Reb 1	76.96 ± 5.34	64.80 ± 8.32
+Reb 10	67.07 ± 4.58	62.16 ± 2.65
+Mir 0.01	69.33 ± 1.87	76.39 ± 9.41
+Mir 0.1	62.79 ± 2.23	76.75 ± 8.72
+Mir 1	76.27 ± 5.42	70.97 ± 7.54
+Mir 10	63.99 ± 2.84	66.21 ± 2.23

MTT reduction assay was performed after treatment of cells with antidepressants [0.01–10 μM: imipramine (Imi)], fluoxetine (Flu), citalopram (Cit), reboxetine (Reb) and mirtazapine (Mir) and staurosporine (St) or doxorubicin (Dox) (0.5 μM) for 24 and 36 h, respectively. Data (*n* = 15) was normalized to vehicle-treated cells (100 %) and expressed as a percent ± SEM * *p* < 0.05 and *** p < 0.001 versus vehicle-treated cells; ^#^ *p* < 0.05 and ^###^ *p* < 0.001 versus St/Dox-treated cells

In RA-SH-SY5Y cells from all tested ADs (0.01–10 μM) only Flu (10 μM) significantly decreased cell viability after 48 but not 24 h of treatment (Sup. Table 1). Moreover, all tested ADs were ineffective in preventing the cell death induced by St (0.5 μM for 24 h) and Dox (1 μM for 48 h) (Sup. Table 1). For Imi (10 μM) and Flu (10 μM) we showed a significant increase in cell death induced by St or Dox by about 20–30 % and 30–40 %, respectively (Sup. Table 1).

### Tianeptine Attenuated the St- and Dox-Evoked DNA Fragmentation in Cortical Neurons and in RA-SH-SY5Y Cells

**Fig. 3:**
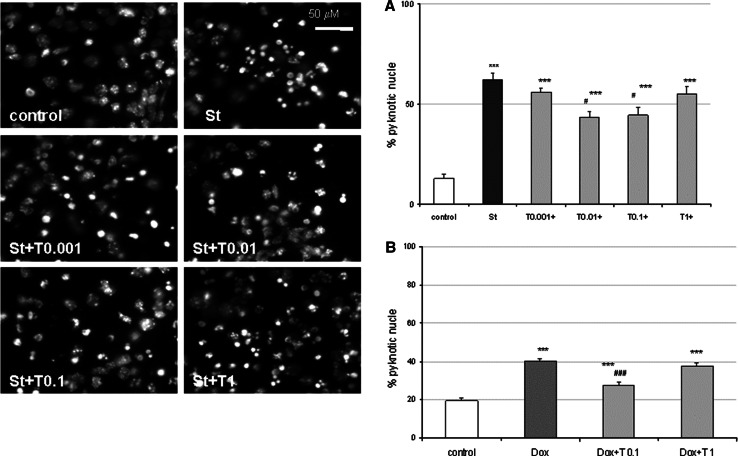
Tianeptine attenuates staurosporine (St)- and doxorubicin (Dox)-induced DNA fragmentation measured by Hoechst 33342 staining. (*Left panel*) Microphotographs from Hoechst 33342 staining of cortical neurons treated with tianeptine (T: 0.001–1 μM) and St (0.5 μM) for 24 h. **a** and **b** Histograms of number of cells with fragmented nuclei after treatment of cortical neurons with tianeptine (T: 0.001–1 μM) and St (0.5 μM) or T (01 and 1 μM) and Dox (0.5 μM) for 24 or 36 h, respectively. Uniformly stained nuclei were scored as healthy, viable nuclei while those with condensed or fragmented nuclei were identified as damaged. The number of cells with nuclei having normal and pyknotic morphology was counted in six randomly chosen fields per a cover slip (150–200 cells); two cover slips per condition from three separate experiments were evaluated. The data were calculated as a percentage of pyknotic nuclei compared to the total number of cells per one field and presented in histograms as the mean ± SEM ***p < 0.001 versus vehicle-treated cells; ^#^ *p* < 0.05 and ^###^ *p* < 0.001 versus St/Dox-treated cells

**Fig. 4:**
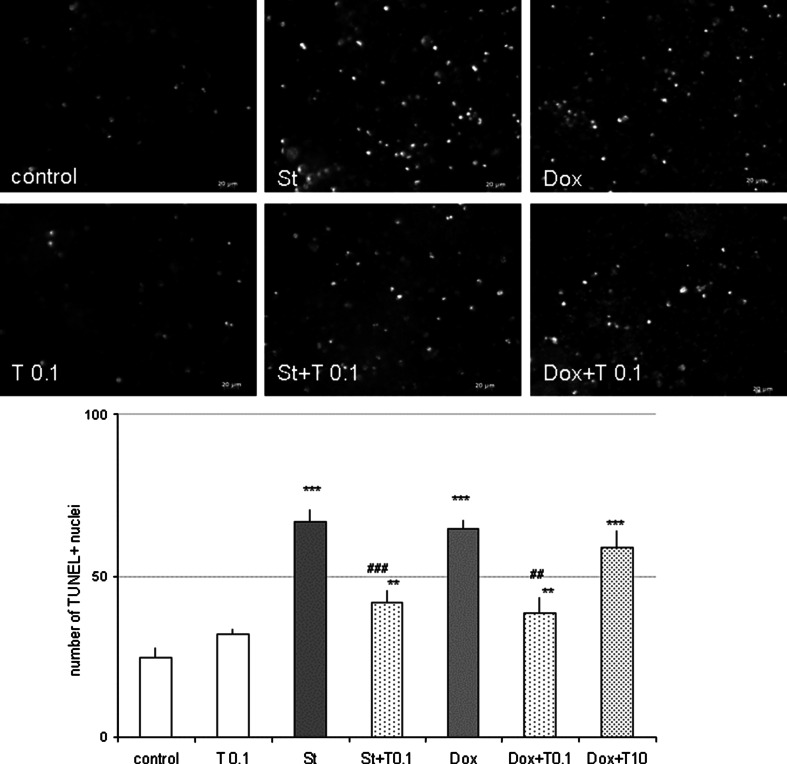
Tianeptine decreases number of apoptotic nuclei estimated by TUNEL assay. (*Upper panel*) Microphotographs from TUNEL labeling of cortical neurons treated with tianeptine (T: 0.1 μM) and staurosporine (St, 0.5 μM) or doxorubicin (Dox, 0.5 μM) for 24 and 36 h, respectively. (*Bottom panel*) Histograms of TUNEL-positive nuclei after treatment of cortical neurons with tianeptine (T: 0.1 and 10 μM) and St (0.5 μM) or Dox (0.5 μM) for 24 or 36 h, respectively. Apoptotic nuclei were counted on recorded images from six randomly chosen fields per a cover slip, two cover slips per condition from three separate experiments and are shown as the mean ± SEM of apoptotic nuclei per one field. **p < 0.01 and ***p < 0.001 versus vehicle-treated cells; ^##^ *p* < 0.01 and ^###^ *p* < 0.001 versus St/Dox-treated cells

Next, the neuroprotection mediated by Tian was evaluated based on the level of DNA changes evoked by St and Dox. Data from Hoechst 33342 staining showed that Tian decreased the number of pyknotic nuclei induced by St and Dox in cortical neurons (Fig. 3) and in RA-SH-SY5Y cells (S. Fig. 3) at the same concentrations in which this drug revealed neuroprotective action in the cell viability tests. Since Hoechst 3342 staining does not clearly distinguish the necrotic and apoptotic morphology of the nucleus, we employed the TUNEL assay as a marker for apoptotic DNA fragmentation. In cortical neurons, we showed the attenuating effect of Tian (0.1 μM) on the number of TUNEL-positive nuclei increased by St and Dox after 24 and 36 h of treatment, respectively (Fig. 4).

**Table 2:** The effects of tianeptine on St- and Dox-induced caspase-3 activity in 7 DIV cortical neurons

	Caspase-3 activity (% control)
Control	100.00 ± 6.27
Tian 10	99.20 ± 6.45
St	186.17 ± 5.95***
+AC-DEVD-CHO	73.52 ± 11.83^###^
+T 0.001	188.77 ± 3.70
+T 0.01	171.70 ± 4.65
+T 0.1	176.76 ± 5.11
+T 1	173.28 ± 3.23
+T 10	172.33 ± 3.40
Dox	174.19 ± 4.30***
+AC-DEVD-CHO	94.59 ± 14.29^###^
+T0.1	176.27 ± 3.46
+T 1	172.10 ± 5.52
+T 10	183.82 ± 3.62

The caspase-3 activity was measured after treatment of cortical cells with tianeptine (T: 0.001–10 μM), AcDEVD-CHO (10 μM) and staurosporine (St, 0.5 μM) or doxorubicin (Dox, 0.5 μM) for 14 (St) and 24 (Dox) hr, respectively. Data was normalized to vehicle-treated cells (100 %) and expressed as a percent ± SEM. *** p < 0.001 versus vehicle-treated cells; ^###^ *p* < 0.001 versus St/Dox-treated cells

### Tianeptine-Mediated Neuroprotection in Cortical Neurons and in RA-SH-SY5Y Cells is Caspase-3-Independent

**Fig. 5:**
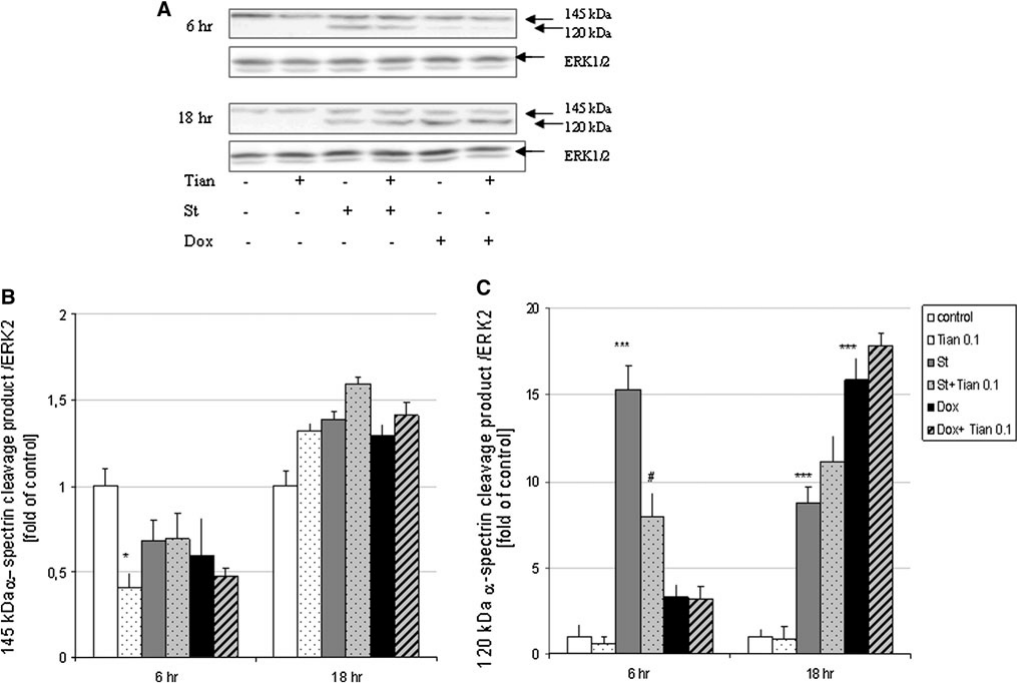
**a** Western blot analysis of spectrin α II cleavage products 145 and 120 kDa induced by calpains and caspase-3, respectively. Cortical neurons were treated with tianeptine (Tian 0.1 μM) and staurosporine (St, 0.5 μM) or doxorubicin (Dox, 0.5 μM) for 6 and 18 h. 145 and 120 kDa spectrin α II cleavage products and ERK2 protein levels were measured in whole cell lysates. **b** and **c** Histograms showing the quantification of Western blots using MultiGauge v.3 Software. The band intensity of spectrin α II cleavage products (145 and 120 kDa) was normalized to ERK2 level (protein load control) and calculated as fold change of control for each blot. Data from duplicate determinations in three independent experiments were shown as the mean ± SEM *p < 0.05 and ***p < 0.001 versus vehicle-treated cells; ^#^ *p* < 0.05 versus St-treated cells

In primary cortical neurons by using the biochemical assay for assessment of caspase-3 activity with specific colorimetric substrate Ac-DEVD-pNa, we showed that the St- and Dox-induced caspase-3 activity was reduced by the caspase-3 inhibitor, AcDEVD-CHO (10 μM) but not by Tian (0.001–10 μM) after 14 and 24 h of treatment, respectively (Table 2). The lack of influence of Tian on caspase-3 activity was also confirmed indirectly by Western Blot analysis of 120 kDa spectrin α ΙΙ cleavage product specifically cleaved by caspases after 18 h of treatment with Tian (0.1 μM) and St and Dox (Fig. 5a, c). However, we noticed some attenuation in the 120 kDa product after 6 h of treatment with Tian (0.1 μM) and St when compared to cells treated only with St (Fig. 5a, c).

In RA-SH-SY5Y cells, we did not find any significant attenuation of St- and Dox-induced caspase-3 activity after 14 and 24 h of treatment, respectively, (S. Table 2) as was confirmed by using biochemical assay with fluorescent substrate Ac-DEVD-AMC.

We also analyzed the 145 kDa spectrin α II cleavage product which is specifically cleaved by calpains, a Ca^2+^-dependent proteases which could be involved in neuronal cell death. We observed only slight increase (not significant) in 145 kDa product after 18 h, but not 6 h of treatment with St and Dox and that effect was not changed by Tian (0.1 μM) (Fig. 5b). After 6 h of treatment, we observed the attenuation by Tian (0.1 μM) in 145 kDa product when compared to control cells (Fig. 5b).

### The Neuroprotection Mediated by Tianeptine in Neuronal Cells is Reduced by PI-3K/Akt and MAPK/ERK1/2 Inhibitors

**Fig. 6:**
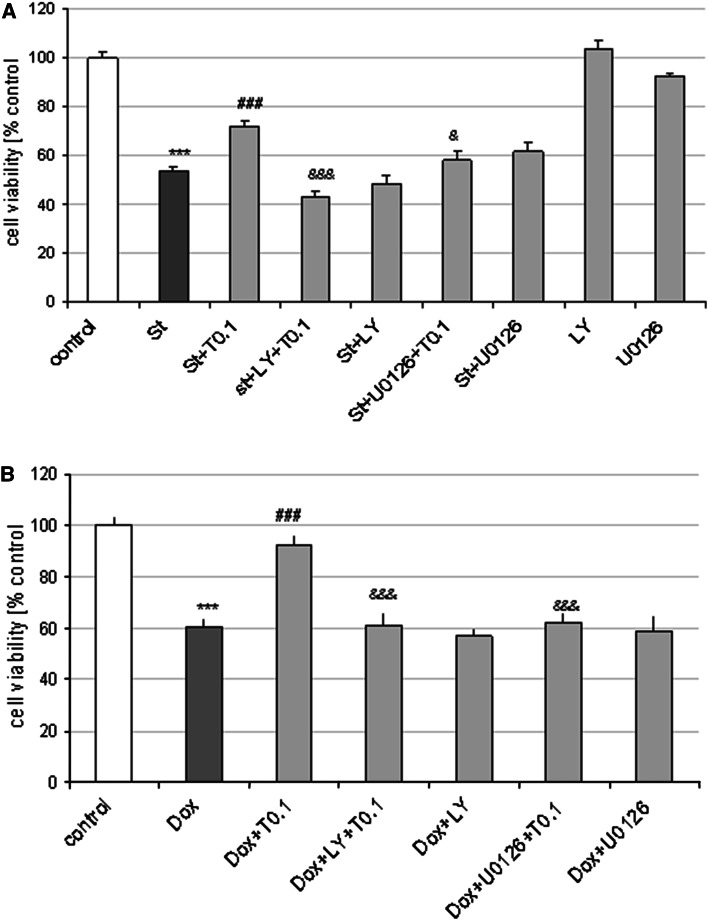
Neuroprotective effects of tianeptine (Tian) are blocked by inhibitors of PI3-K/Akt and MAPK/ERK1/2 pathways. Cell viability after treatment of cortical neurons with PI3-K/Akt inhibitor, LY294002 (10 μM) and MAPK/ERK1/2 inhibitor, U0126 (10 μM) and Tian (0.1 μM) and **a** staurosporine (St, 0.5 μM) or **b** doxorubicin (Dox, 0.5 μM) for 24 or 36 h, respectively. ***p < 0.001 versus vehicle-treated cells; ^###^ *p* < 0.001 versus St/Dox- treated cells; ^&^ *p* < 0.05 and ^&&&^ *p* < 0.001 versus St/Dox + Tian-treated cells

In order to verify the engagement of pro-survival PI3-K/Akt and MAPK/ERK1/2 pathways in neuroprotection mediated by Tian, we used pharmacological inhibitors. The MAPK/ERK1/2 inhibitor, U0126 (10 μM) and the PI3-K/Akt inhibitor, LY294002 (10 μM) attenuated the protection mediated by Tian (0.1 μM) against St- and Dox-induced cell damage in cortical neurons (Fig. 6a, b) as well as in RA-SH-SY5Y cells (S. Fig. 4) at the same time having no influence on the extent of cell damage induced by the used cell death-inducing agents.

**Fig. 7:**
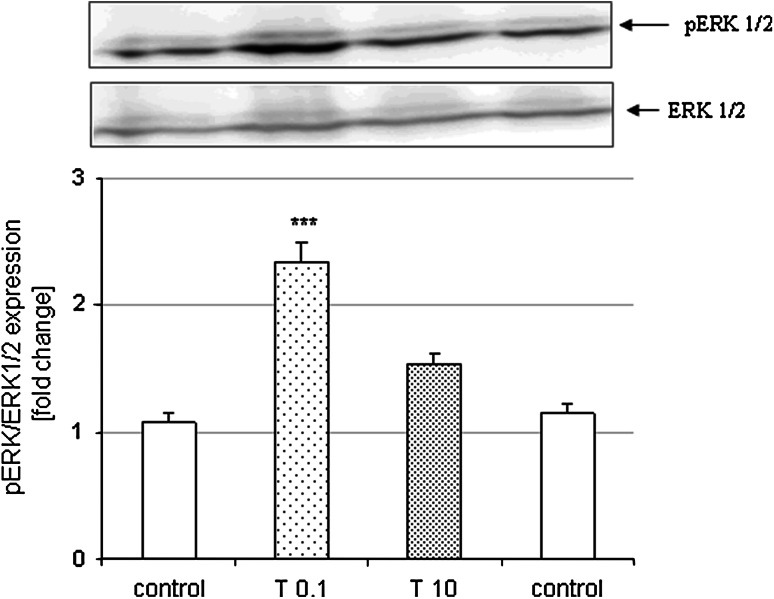
(*Upper panel*) Representative *blots* showing the phosphorylated (pERK) and total form of ERK 1/2 after 6 h of treatment with Tian (0.1 and 10 μM). *Histograms* show the quantification of Western blots using MultiGauge v.3 Software. The band intensity of pERK was normalized to ERK 1/2 level and calculated as fold change of control for each blot. Data from duplicate determinations in three independent experiments were shown as the mean ± SEM ***p < 0.001 versus vehicle-treated cells

Performing Western blot analysis of cortical neurons treated for 6 h with Tian we demonstrated that low concentration of Tian (0.1 μM) but not higher one (10 μM) increased the pERK level when compared to vehicle-treated cells (Fig. 7).

### Tianeptine-Evoked Neuroprotection was Inhibited by the Necroptosis Inhibitor, Necrostatin-1

**Fig. 8:**
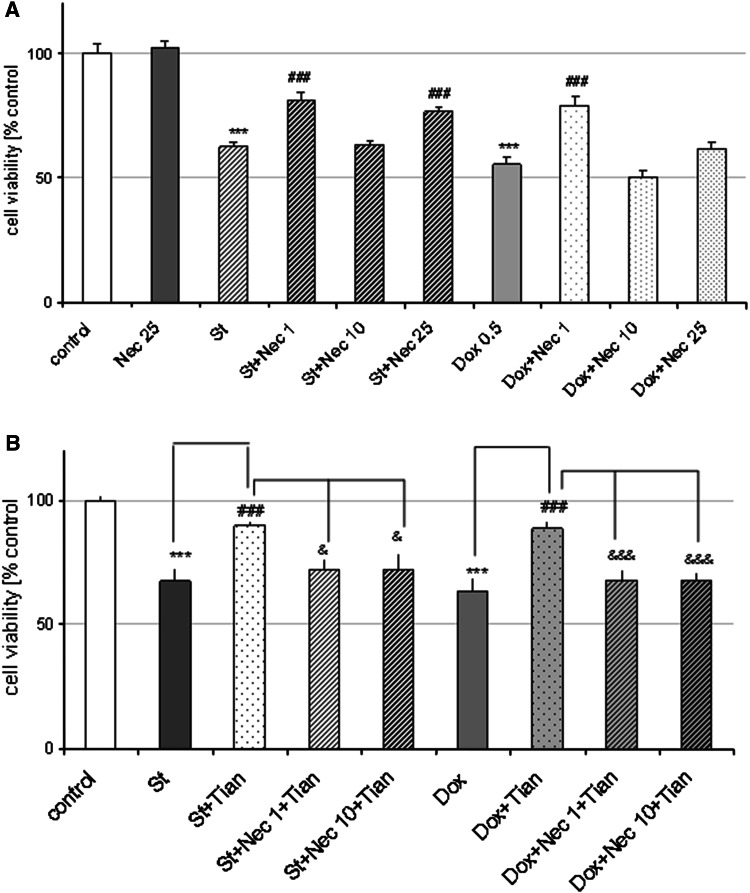
Necrostatin-1 (Nec-1) is neuroprotective against St- and Dox-evoked cell damage as well diminishes the protection mediated by Tian. **a** Cell viability after treatment of cortical neurons with inhibitor of necroptosis, Nec-1 (1-25 μM) and staurosporine (St, 0.5 μM) and doxorubicin (Dox, 0.5 μM) for 24 and 36 h, respectively. **b** Cell viability after treatment of cortical neurons with inhibitor of necroptosis, Nec-1 (1 and 10 μM) and Tian (0.1 μM) and staurosporine (St, 0.5 μM) and doxorubicin (Dox, 0.5 μM) for 24 and 36 h, respectively. Data were normalized to the absorbance in the vehicle-treated cells (100 %) and expressed as a percent of the control ± SEM established from *n* = 5 wells per one experiment from four separate experiments. ***p < 0.001 versus vehicle-treated cells; ^#^ *p* < 0.05 and ^###^ *p* < 0.001 versus St/Dox- treated cells; ^&^ *p* < 0.05 and ^&&&^ *p* < 0.001 versus St/Dox + Tian-treated cells

Since we excluded the participation of caspase-3 in neuroprotection mediated by Tian, in the next experiments we checked the engagement of a caspase-3-independent form of cell death, necroptosis, by the use of the inhibitor of this programmed necrotic cell death, necrostatin-1 (Nec-1). We showed that Nec-1 at specific concentrations served protection against St-(Nec-1 1 μM and 25 μM, but not 10 μM) and Dox-(Nec-1 1 μM but not 10 and 25 μM) evoked cell damage (Fig. 8a). Moreover, using a protective and non-protective concentrations of Nec-1 (1 and 10 μM, respectively) we demonstrated inhibition by this agent at both concentrations of the protection mediated by Tian (0.1 μM) against St- and Dox-evoked neuronal cell damage (Fig. 8b).

### Predominant Neuroprotective Effect of Various Antidepressants in Primary Glia Cells When Compared to Neuronal Cultures

**Fig. 9:**
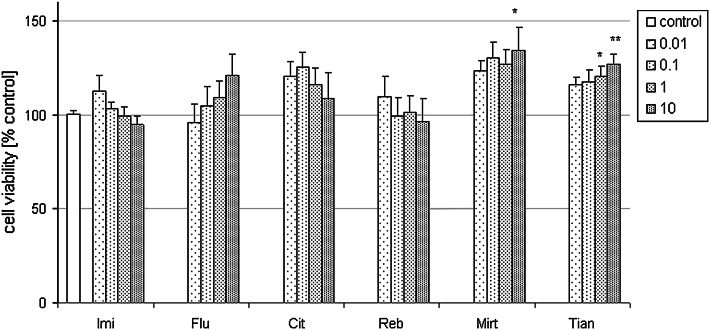
Effect of all tested antidepressants (ADs) when given alone on cell viability of glia cultures. 4-weeks old glia cells were treated with ADs: imipramine (Imi), fluoxetine (Flu), citalopram (Cit), reboxetine (Reb), mirtazapine (Mirt), and tianeptine (Tian) at concentrations from 0.01 to 10 μM. Cell viability was estimated via MTT reduction method and data were normalized to the absorbance in the vehicle-treated cells (100 %) and expressed as a percent of the control ± SEM established from *n* = 5 wells per one experiment from three separate experiments. *p < 0.05 and ***p < 0.01 versus vehicle-treated cells

**Fig. 10:**
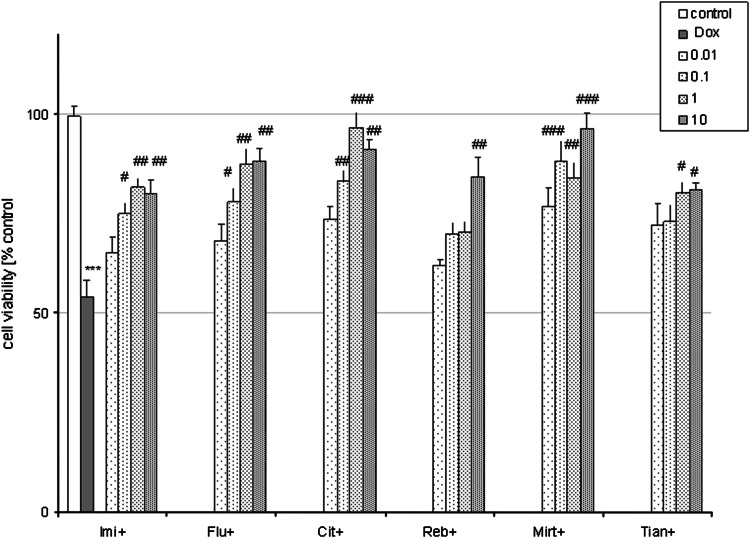
All tested antidepressants (ADs) are protective against doxorubicin (Dox)-evoked cell damage in glia cells. 4-weeks old glia cells were co-treated with ADs: imipramine (Imi), fluoxetine (Flu), citalopram (Cit), reboxetine (Reb), mirtazapine (Mirt), and tianeptine (Tian) at concentrations from 0.01 to 10 μM and doxorubicin (Dox, 1 μM for 48 h). Cell viability was estimated via MTT reduction method and data were normalized to the absorbance in the vehicle-treated cells (100 %) and expressed as a percent of the control ± SEM established from *n* = 5 wells per one experiment from three separate experiments. ***p < 0.001 versus vehicle-treated cells; ^#^ *p* < 0.05, ^##^ *p* < 0.01, and ^###^ *p* < 0.001 versus Dox-treated cells

In glia cells, various tested ADs (Imi, Flu, Cit, Reb, Mirt, Tian) in a wide concentration range (0.01–10 μM) when given alone for 24 h had no influence on the basal LDH release (not shown) but a significant increase in cell viability was found for Mirt (10 μM) and Tian (1 and 10 μM)) as compared to the control cells (Fig. 9). Doxorubicin (1 μM for 48 h) evoked about 50 % reduction in glia cell viability as was measured by MTT reduction assay (Fig. 10). Moreover, all tested ADs in various concentrations: Imi (0.1–10 μM), Flu (0.1–10 μΜ), Cit (0.1–10 μM), Reb (10 μM), Mirt (0.1–10 μM), and Tian (1 and 10 μM) were protective against cell death induced by Dox (1 μM) (Fig. 10).

## Discussion

The results of the present study demonstrated that (1) from the group of six tested ADs with differential mechanisms of action (Imi, Flu, Cit, Reb, Mirt, Tian) only Tian was found to be partially protective against St- and Dox-evoked neuronal cell death, however, in glia cells all tested AD drugs provided significant protection against Dox-evoked cell death, (2) neuroprotection mediated by Tian was accompanied by the attenuation of apoptotic DNA fragmentation, although this effect was caspase-3-independent, (3) protection evoked by Tian was reduced by inhibitors of PI3-K/Akt and MAPK/ERK1/2 pathways, and (4) the inhibitor of necroptosis, necrostatin-1 completely blocked the Tian-mediated protection.

Our findings add to data on neuroprotective effects of Tian and extend them to new models of apoptosis (St and Dox), where Tian showed a significant protection. Moreover, in our study, the protective effect of Tian was accompanied with attenuation of DNA fragmentation but rather was not connected with changes in caspase-3 activity as was measured on an in vitro assay with a standard substrate.

Most previous reports showing neuroprotective action of Tian in various in vivo and in vitro experimental settings employed the chronic schedule of drug administration (Lucassen et al. 2004; Plaisant et al. 2003). In our study, we found protection after a single administration of Tian in a model of neuronal injury not directly connected with glutamate overactivity. This is in line with another report showing protection after a single, but also after 5 day administration of Tian (10 mg/kg i.p.) against brain lesions induced by a combination of NMDA and IL-1β (Plaisant et al. 2003). There are studies showing the anti-apoptotic action of Tian (50 mg/kg p.o., 28 days) which was evidenced by attenuation of Tunel-positive cells in the temporal cortex and hippocampal subgranular zone of animals exposed to chronic psychosocial stress. However, that in vivo study did not address the question of whether this effect was neuronal or glia specific (Lucassen et al. 2004). In contrast to Tian, in our study we did not notice any protection mediated by a single administration of other tested ADs (Imi, Flu, Cit, Reb, Mirt) to primary neurons as well as to neuronally differentiated human neuroblastoma SH-SY5Y cells against St and Dox neurotoxicity. In contrast, previous studies demonstrated neuroprotective properties of various ADs in different models of cell death, however, these effects were found after chronic administration of drugs (reviewed by Drzyzga et al. 2009; McKernan et al. 2009). There are also reports showing that a single administration of some ADs could be protective in various models of cellular injury although these agents were not tested against St- and Dox-evoked cell death in neuronal cells (Chiou et al. 2006; Chung et al. 2011; Nahon et al. 2005; Zhang et al. 2012a). For example, Flu in concentration of 3 μM was found to be neuroprotective against LPS- and MPP + (1-methyl-4-phenylpyridinium)-evoked neurotoxicity in neuronal-glia co-cultures of midbrain neurons, whereas in concentration >10 μM attenuated the St-evoked apoptosis of U937 cells (Nahon et al. 2005; Zhang et al. 2012a). However, in our study, we observed cytotoxic effect of Flu (10 μM) after 24 and 36 h of treatment in primary neurons when in RA-SH-SY5Y cell-damaging effect of Flu (10 μM) was noticed only after 48 but not 24 h of treatment (Table 1; Sup. Table 1). Using higher concentration of Flu (50 μM), we observed highly toxic effect of this drug in both, primary neurons as well in RA-SH-SY5Y cells (data not shown). Moreover, in our study Flu (10 μM) also increased the cell death induced by St and Dox in primary neurons as well in RA-SH-SY5Y cells, whereas Imi (10 μM) elevated the cell death evoked by St and Dox only in RA-SH-SY5Y cells. Thus, the above data add to the reports demonstrating pro-apoptotic action of higher concentrations of some ADs when given alone or in combination with some chemotherapeutics which seems to be dependent on the type of AD drug, the used cells as well as on cell culture conditions (Abdel-Razaq et al. 2011; Argov et al. 2009; Drzyzga et al. 2009; Levkovitz et al. 2005).

In contrast to neuronal cells, in glia cells we demonstrated a protective effect of all tested ADs against cell death induced by Dox. This observation suggests that the previously described neuroprotective effects of various ADs in the in vivo situation could be partly due a result of their influence on glia cells (Drzyzga et al. 2009; Martin et al. 2013; McKernan et al. 2009). The more detailed studies in neuronal-glia cultures showed that the regulating effect of this drug on the pro-inflammatory factors induced by toxin exposure was the key step in the neuroprotective effects of Tian or Flu (Chung et al. 2011; Plaisant et al. 2003; Zhang et al. 2012a). It should be underlined that in our study the protective effect of Tian in neuronal and glia cells differed according to the concentrations used, namely Tian at low concentrations (0.01 an 0.1 μM) was effective in neurons while at higher doses (1 and 10 μM) it was protective in glia cells, which suggests the involvement of different mechanisms. Regarding the protective effect of Mirt and Tian in glia cells against Dox-evoked cell damage, it cannot be excluded that this effect was due to their influence on cell proliferation since these agents when given alone increased the viability of glia cells (Fig. 7). Further evaluation of the mechanisms engaged in ADs-mediated neuroprotection against Dox toxicity would be very important from the clinical point of view since glia cell damage occurs during chemotherapy and concomitantly with neuronal cell damage participate in neurocognitive impairments observed in patients after long-term chemotherapy (Bigotte and Olsson 1983; Moriuchi et al. 1996; O’Farrell et al. 2013; Tarasenko et al. 2012). Regarding the protective effects of Tian in glia cells, it has been shown that Tian (10–100 μM) attenuated the gp120-evoked cell death in glia cells and this effect was connected with attenuation of caspase-3 activity, DNA fragmentation and the influence of this drug on the expression and activity of glutamine synthase (GS), transcription factor NFκB, and nitric oxide system (NO, cNOS, iNOS) (Janda et al. 2011).

Studying the possible mechanism of neuroprotective action of Tian in cortical neurons and RA-SH-SY5Y cells, we excluded the participation of caspase-3 on the basis of performed biochemical assay using caspase-3 specific substrate. We confirmed these results by second method, analysis of 120 KDa cleavage product of spectrin α II induced by caspases, where we did not observe any attenuation of St- or Dox-evoked increase in this product after 18 h of treatment with Tian and pro-apoptotic factors. However, after 6 h of treatment with drugs, we noticed a significant reduction in 120 kDa product in St + Tian group when compared to cell treated only with St. These data suggests that Tian could interfere with the caspase-3 activity transiently which could only delay but not stop the cell death progression induced by St or Dox. In fact, inhibitor of caspase-3 (Ac-DEVD-CHO) was able only partially (about 25 % decrease in LDH level) diminish the cell death induced by St and Dox in cortical neurons as we reported in our previous studies (Leskiewicz et al. 2008; Jantas and Lason 2009). The data obtained in this study suggests that some caspase-3-independent mechanisms could be engaged in Tian neuroprotection. Exploring further the caspase-3-independent targets for the neuroprotective action of Tian, we tested the engagement of necroptosis, a programmed necrotic cell death pathway (Christofferson and Yuan 2010). It is well known that the same harmful stimulus can induce both apoptosis and necroptosis in in vitro and in vivo conditions (Ji et al. 2011; Šimenc and Lipnik-Štangelj 2012; Wang et al. 2012). Necroptosis was firstly discovered in immunology and oncology field, however, recently it is also very often described in relation to neurodegeneration, e.g., in the retinal ischemia–reperfusion injury model, neonatal ischemia, and glutamate toxicity (Chavez-Valdez et al. 2012; Degterev et al. 2005, 2008; Northington et al. 2011; Rosenbaum et al. 2010; Wang et al. 2012; Xu et al. 2007; Zhang et al. 2013). The participation of necroptosis was also reported to be involved in St-evoked cell damage in glia cells and U937 cells as well as in cisplatin-induced kidney injury (Dunai et al. 2012; Šimenc and Lipnik-Štangelj 2012; Tristão et al. 2012). In our study, we showed that the inhibitor of necroptosis, necrostatin-1 (Nec-1) was able to attenuate the extent of cell damage evoked by St and Dox, however the most significant effect was achieved at its low concentration (1 μM) and this effect was comparable with protection mediated by Tian (about 30 %). Moreover, in case of St toxicity, we observed protective effect of Nec-1 25 μM, but not 10 μM. These observations suggest that possible different mechanisms could be involved in Nec-1 mediated neuroprotection al least in St model of neuronal cell death. Interestingly, in our study the protective as well non-protective concentrations of Nec-1 (1 and 10 μM, respectively) inhibited the protection mediated by Tian against St- and Dox-induced cell damage. However, the mechanisms responsible for observed by us effects with Nec-1 needs further investigations starting from the study of activity of receptor interacting protei kinase 1 (RIP1) which is a main player in induction of necroptotic pathway (Christofferson and Yuan 2010; Degterev et al. 2005, 2008). In fact, the involvement of this type of programmed cell death in neurodegeneration and neuroprotection field it still at its early years of investigation and there are plenty of unresolved questions, especially RIP1 downstream executioners has not been identified yet (Christofferson and Yuan 2010). Nevertheless, it has been shown that necroptosis could be involved in protection mediated by curcumin against iron induced neurotoxicity in primary cortical neurons (Dai et al. 2013).

The last aspect of our study was demonstration that the Tian-mediated protection against St and Dox toxicity in cortical neurons and in RA-SH-SY5Y cells was attenuated by inhibitors of MAPK/ERK1/2 and PI3K/Akt pro-survival pathways. This suggests the existence of a common upstream step which possibly is regulated by Tian and consequently leads to the activation of PI3-K/Akt and MAPK/ERK1/2. The involvement of the above pro-survival kinase pathways in the protective effects of various potentially neuroprotective drugs was firmly evidenced (Gabryel et al. 2002; Hetman and Gozdz 2004; Sawe et al. 2008; Zhu et al. 2004). In relation to ADs, it has been shown that Flu could induce MAPK/ERK1/2 and PI3-K/Akt pathways in astrocytes whereas moclobemide (50 μM) increased pERK level in neural stem cells (McKernan et al. 2009). It is highly possible that the activation of pro-survival intracellular pathways by ADs could be the effect of action of neurotrophic factors (e.g., BDNF, GDNF) the increase of which has been reported in some forebrain structures after acute and chronic ADs treatment including Tian (Chu et al. 2010; Della et al. 2012b, 2013; Jang et al. 2009; McKernan et al. 2009; Saarelainen et al. 2003; Sairanen et al. 2005, 2007). In our study, we confirmed the participation of activation of ERK 1/2 pathway in neuroprotective effects of Tian by showing the increase in pERK protein level after 6 h of treatment with low (0.1 μM) but not higher (10 μM) concentration of Tian. These data strengthen the hypothesis proposed by Xu et al. (2003), who suggested that some ADs affect pro-survival pathways only in lower concentrations, not in higher one. These authors demonstrated in in vivo situation that the neuroprotective effects of ADs may be limited to a dose that, when exceeded, provides no neuroprotection or even exerts cytotoxic effect. This was shown in study with chronic (21 days) amitriptyline or venlafaxine i.p. administration to male Sprague–Dawley rats where low dose (5 mg/kg), but not higher (10 mg/kg) of both drugs increased BDNF in hippocampus (Xu et al. 2003). Interestingly, in our very recent paper we found that various ADs, including Tian, only in lower concentrations but not in higher one prevented the anti-proliferative effect of dexamethasone in human neuroblastoma SH-SY5Y cells and this effect was connected with their influence on ERK1/2 activity (Leskiewicz et al. 2013).

Summing up, we demonstrated that from the group of the tested ADs with differential mechanisms of action (Imi, Flu, Cit, Reb, Mirt, Tian) only Tian was found to be protective in vitro against St- and Dox-evoked neuronal cell death. However, in glia cells all tested ADs were protective against Dox-evoked cell death. Next, we showed that neuroprotection mediated by Tian was accompanied by the attenuation of apoptotic DNA fragmentation, although this effect was rather caspase-3-independent. Finally, the protection mediated by Tian was attenuated by inhibitors of PI3-K/Akt and MAPK/ERK1/2 pathways as well as by the necroptosis inhibitor, necrostatin-1 which points to involvement of these processes in the molecular mechanism of neuroprotective action of Tian.

## Electronic supplementary material

Below is the link to the electronic supplementary material.

Supplementary material 1 (DOC 712 kb)

## Abbreviations

ADs: Antidepressant drugs
AIF: Apoptosis-inducing factor
Cit: Citalopram
Dox: Doxorubicin
Flu: Fluoxetine
Imi: Imipramine
LDH: Lactate dehydrogenase
Mirt: Mirtazapine
MTT: 3-[4,5-Dimethylthiazol-2-yl]-2,5-diphenyltetrazolium bromide
RA-SH-SY5Y: Retinoic acid-differentiated SH-SY5Y cells
Reb: Reboxetine
St: Staurosporine
Tian: Tianeptine
