# Supplementary material for: The Predominant Protective Effect of Tianeptine Over Other Antidepressants in Models of Neuronal Apoptosis: The Effect Blocked by Inhibitors of MAPK/ERK1/2 and PI3-K/Akt Pathways
Source: Neurotox Res. 2013 Oct 9;25(2):208–25. doi: 10.1007/s12640-013-9430-3 (PMC3889694; doi:10.1007/s12640-013-9430-3)

**S.Tab.1.** The effects of antidepressants on St- and Dox-evoked cell death in RA-SH-SY5Y

|  | cell viability - **St**  [% control] | cell viability - **Dox**  [% control] |
| --- | --- | --- |
| control | 100.03 ± 3.52 | 99.96 ± 2.62 |
| Imi 10 | 92.97 ± 3.95 | 92.97 ± 7.02 |
| Flu 10 | 109.43 ± 11.12 | 84.25 ± 3.95 * |
| Cit 10 | 104.44 ± 3.78 | 95.50 ± 5.28 |
| Reb 10 | 107.86 ± 4.39 | 97.07 ± 2.62 |
| Mirt 10 | 102.05 ± 5.12 | 96.87 ± 7.55 |
| St/Dox | 66.67 ± 4.48 *** | 61.38 ± 3.30 *** |
| + Imi 0.01  + Imi 0.1  + Imi 1  + Imi 10 | 64.00 ± 6.52  61.07 ± 7.40  52.26 ± 5.22  34.93 ± 13.66 # | 50.41 ± 4.16  56.78 ± 2.67  52.260 ± 2.84  45.09 ± 3.26 # |
| + Flu 0.01  + Flu 0.1  + Flu 1  + Flu 10 | 64.47 ± 16.98  69.15 ± 8.55  58.47 ± 19.22  23.98 ± 2.79 ### | 54.54 ± 3.36  60.81 ± 2.26  64.80 ± 4.01  28.87 ± 3.01 ### |
| + Cit 0.01  + Cit 0.1  + Cit 1  + Cit 10 | 76.33 ± 13.26  73.67 ± 3.14  68.49 ± 4.22  52.38 ± 4.83 | 54.96 ± 3.73  67.99 ± 2.44  65.17 ± 3.94  52.26 ± 3.95 |
| + Reb 0.01  + Reb 0.1  + Reb 1  + Reb 10 | 86.81 ± 15.98  83.11 ± 15.16  64.89 ± 2.98  46.51 ± 13.65 | 62.53 ± 2.57  69.49 ± 3.10  63.17 ± 2.06  54.64 ± 2.38 |
| + Mir 0.01  + Mir 0.1  + Mir 1  + Mir 10 | 72.53 ± 7.11  78.27 ± 7.44  68.40 ± 11.21  51.20 ± 8.83 | 55.95 ± 3.83  63.26 ± 2.28  65.06 ± 2.41  67.95 ± 2.98 |

MTT reduction assay was performed after treatment of cells with antdepressants (0.01-10 μM: imipramine (Imi), fluoxetine (Flu), citalopram (Cit), reboxetine (Reb) and mirtazapine (Mir) and St (0.5 μM) or Dox (0.5 μM) for 24 and 48 hr, respectively. Data (n=15) was normalized to vehicle-treated cells (100 %) and expressed as a percent ± S.E.M. ^*^p<0.05 and ^***^p<0.001 vs vehicle-treated cells; ; ^#^p<0.05 and ^###^p<0.001 vs St/Dox- treated cells

**S.Tab.2.** The effects of tianeptine on St-induced caspase-3 activity in RA-SH-SY5Y cells

|  | caspase-3 activity  [RFU/mg protein] |
| --- | --- |
| control | 61.82 ± 6.61 |
| Tian 10 | 64.13 ± 2.29 |
| St  +Ac-DEVD-CHO  + T 0.01  + T 0.1  + T 1  + T 10 | 328.65 ± 4.07 ***  27.89 ± 4.41 ###  258.30 ± 34.82  274.26 ± 33.39  312.15 ± 5.09  315.67 ± 11.52 |
| Dox  +Ac-DEVD-CHO  + T 0.01  + T 0.1  + T 1  + T 10 | 266.27 ± 30.40 ***  30.57 ± 3.41 ###  232.48 ± 38.58  268.99 ± 26.91  296.20 ± 24.70  292.40 ± 29.64 |

The caspase-3 activity was measured after treatment of SH-SY5Ycells with tianeptine (T: 0.01-10 μM), AcDEVD-CHO (10 μM) and staurosporine (St, 0.5 μM) or doxorubicin (Dox) for 14 hr and 24 hr respectively. Data was demonstrated as relative fluorescence units (RFU)/mg protein± S.E.M. ^***^p<0.001 vs vehicle-treated cells; ^###^p<0.001 vs St- treated cells.

**Figure legends**

**S.Fig.1.** Microphotographs from alive imaging (CalceinAM staining) of cortical neurons treated with tianeptine (T:0.01; 0.1 and1 μM) and staurosporine (St, 0.5 μM) for 24 hr. scale bar 25 μm.

**S.Fig.2.** Tianeptine is neuroprotective against staurosporine (St)- and doxorubicin (Dox)- evoked cell damage in retinoic acid-differentiated SH-SY5Y (RA-SH-SY5Y) cells. (A and B) RA-SH-SY5Y cells were co-treated with tianeptine (T:0.01-10 μM) and St (0.5 μM) for 24 hr. Next, LDH level (A) was measured in the culture medium from the treated cells and cell viability was estimated in cells via MTT reduction method (B). (C and D) RA-SH-SY5Y cells were co-treated with tianeptine (T:0.01-10 μM) and Dox (1 μM) for 48 hr. Next, LDH level (C) was measured in the culture medium from the treated cells and cell viability was estimated in cells via MTT reduction method (D). (A-D) The data from the biochemical tests were normalized to the absorbance in the toxin-treated cells (100%) and expressed as a percent of the control±S.E.M. established from n=5 wells per one experiment from 3 separate experiments. ^***^p<0.001 vs vehicle-treated cells; ^#^p<0.05 and ^###^p<0.001 vs St/Dox- treated cells.

**S.Fig.3.** Tianeptine attenuates staurosporine (St)- and doxorubicin (Dox)- induced DNA fragmentation in RA-SH-SY5Y cells. Microphotographs and histograms show RA-SH-SY5Y stained with Hoechst 33342 dye after treatment of cells with tianeptine (T: 0.1 μM) and St (0.5 μM) for 24 hr or Dox (1 μM) for 48 hr. Uniformly stained nuclei were scored as healthy, viable nuclei while those with condensed or fragmented nuclei were identified as damaged. The number of cells with nuclei having normal and pyknotic morphology was counted in six randomly chosen fields per a cover slip (150-200 cells); two cover slips per condition from 3 separate experiments were evaluated. The data were calculated as a percentage of pyknotic nuclei compared to the total number of cells per one field and presented in histograms as the mean±S.E.M. ^***^p<0.001 vs vehicle-treated cells; ^###^p<0.001 vs St/Dox- treated cells.

**S.Fig.4.** Protective effects of tianeptine (Tian) in RA-SH-SY5Y cells are blocked by inhibitors of PI3-K/Akt and MAPK/ERK1/2 pathways. Cell viability was measured after treatment of RA-SH-SY5Y cells with PI3-K/Akt inhibitor, LY294002 (10 μM) and MAPK/ERK1/2 inhibitor, U0126 (10 μM) and Tian (0.1 μM) and (A) staurosporine (St, 0.5 μM) or (B) doxorubicin (Dox, 0.5 μM) for 24 or 36 hr, respectively. ^***^p<0.001 vs. vehicle-treated cells; ^#^p<0.05 and ^###^p<0.001 vs. St/Dox- treated cells; ^&^p<0.05 and ^&&&^p<0.001 vs. St/Dox+Tian-treated cells.


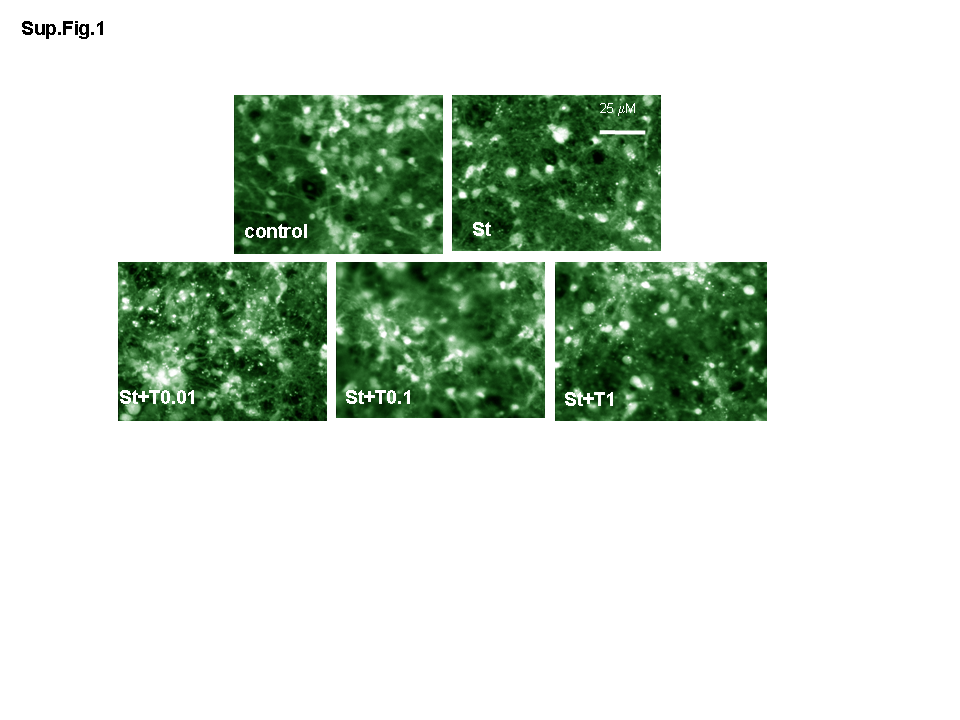


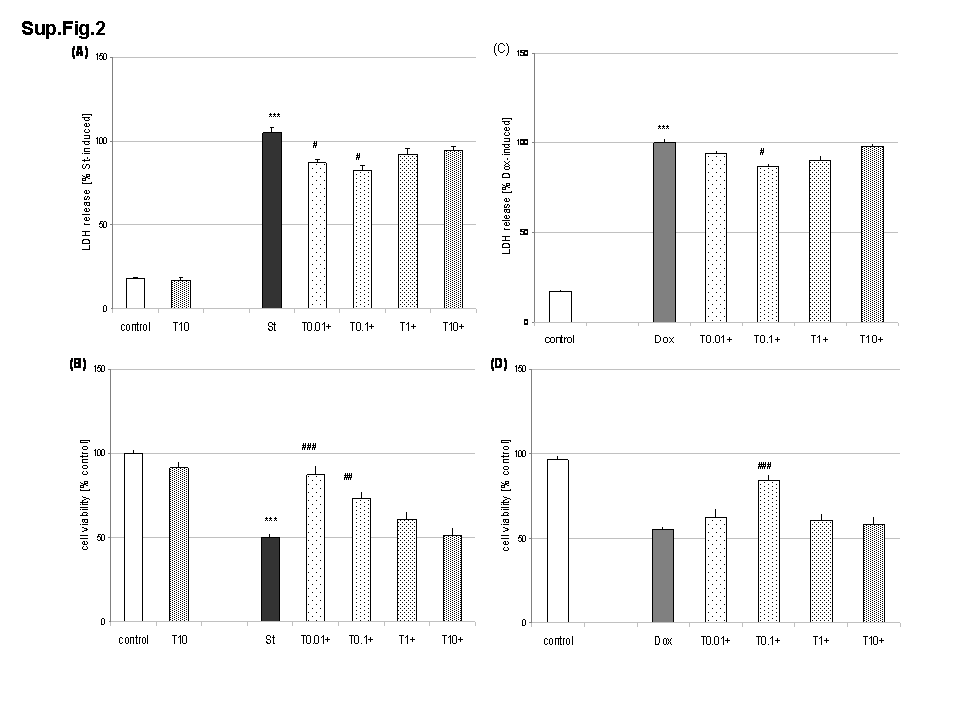


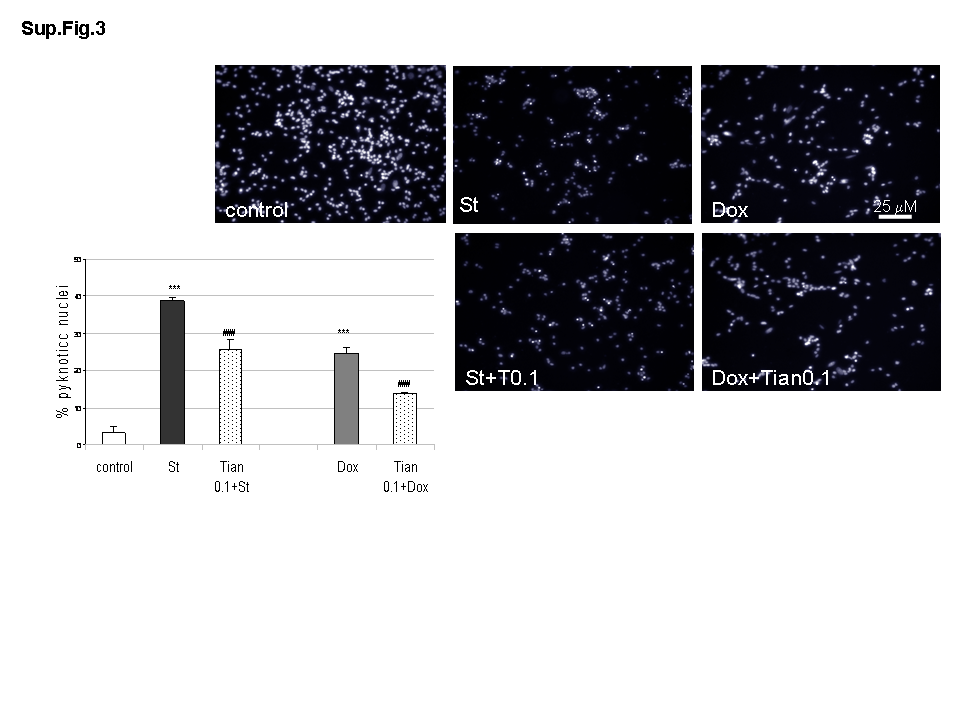


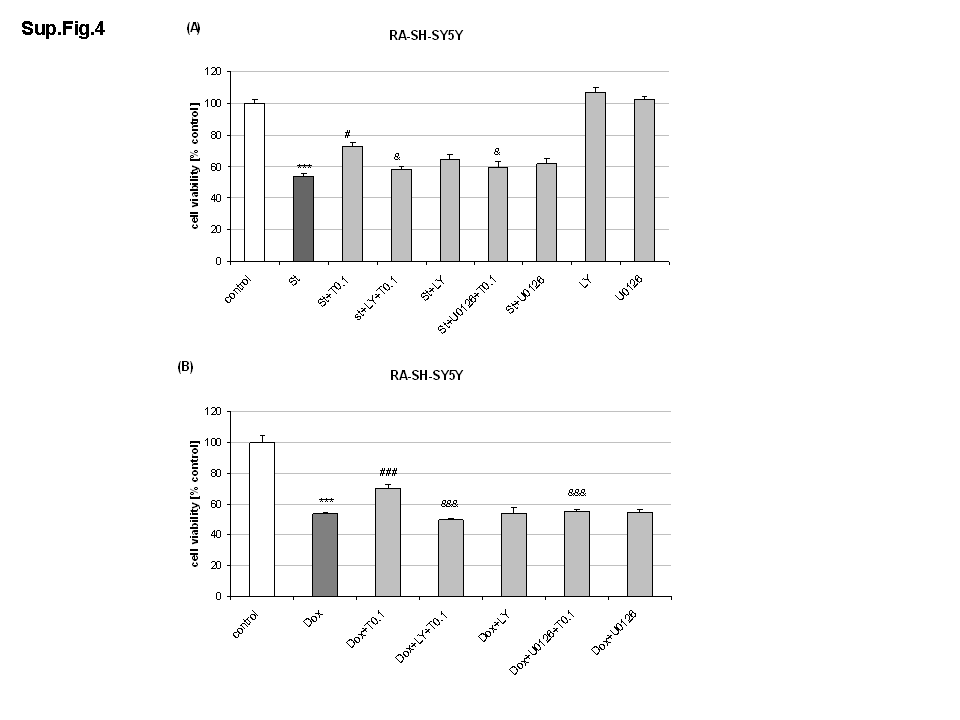

Supplement: Supplementary file 1 — Supplementary material 1 (DOC 712 kb) [file 12640_2013_9430_MOESM1_ESM.doc]
